# Molecular Epidemiology of Human Metapneumovirus in Kilifi, Coastal Kenya, 2016-2017 and 2021-2024

**DOI:** 10.12688/wellcomeopenres.25865.2

**Published:** 2026-07-02

**Authors:** Dorcas Okanda, Samuel O. Odoyo, Hillary Kiprono, Esther Katama, Grace Maina, Martin Mutunga, Joyce Nyiro, Charles N Agoti, George Githinji

**Affiliations:** 1 Epidemiology and Demography, Centre for Geographic Medicine Research Coast, Kilifi, Kilifi County, Kenya; 2 School of Public Health, Pwani University, Kilifi, Kilifi County, Kenya; 3 Department of Biochemistry and Biotechnology, Pwani University, Kilifi, Kilifi County, Kenya

**Keywords:** hMPV, A2c-111nt-dup, whole genome sequencing, Oxford Nanopore Technologies, Kilifi, Kenya

## Abstract

**Background:**

Human metapneumovirus (hMPV) is a major contributor of acute respiratory infections (ARI) in childhood and vulnerable adults. It comprises two antigenically distinct lineages (A and B), with multiple sub-lineages. Genomic analyses of hMPV strains enable monitoring of viral evolution and transmission to inform future interventions but remain underutilized in Africa.

**Methods:**

We generated 52 near-complete hMPV genomes from respiratory samples collected in Kilifi, Coastal Kenya, using a tiled-amplicon approach and Oxford Nanopore Technologies sequencing. These samples had been identified as hMPV positive by quantitative PCR during (a) a multi-facility outpatient ARI surveillance in nine health facilities in Kilifi between 2016 and 2017, and 2021 to 2023 and (b) a community-based respiratory infection cohort surveillance study between 2023-2024 that sampled enrolled participants irrespective of symptom status.

**Results:**

Of the 192 positive samples analyzed from the two studies, children under 5 years accounted for most hMPV cases (134/186, 72%). 52 samples were sequenced (>70% genome coverage), and hMPV-A (27/52, 53.8%) and hMPV-B (25/52, 46.2%) lineages were identified. The recovered sequences mapped into sub-lineages A2c (27/52, 53.8%), B1 (12/52, 21.2%), and B2b (13/52, 25%). A shift in the predominant sub-lineage was observed from B2b (2016) to B1 (2021), and finally to A2c-wild type (2023). In February 2021, for the first time, we detected a single A2c strain with a 111-nucleotide duplication in the G gene among Kenyan samples.

**Conclusion:**

Our study expands the global nucleotide sequence database for hMPV by adding new whole-genome sequences from Kenya collected over the last decade. It highlights the ongoing replacement of locally predominant hMPV lineages and the importation and local transmission of globally circulating strains. These findings underscore the importance of sustained hMPV genomic surveillance to detect emerging variants and monitor lineage circulation patterns that may impact viral transmission, molecular detection, and future control measures.

## Plain Language summary

Human metapneumovirus (hMPV) infection can result in mild-to-severe acute respiratory illness, especially in young children, the elderly and individuals with weakened body defenses. Analyzing the genetic code of hMPV can enhance understanding on how this virus spreads and adapts to host populations over time. However, such investigations are scarce in Africa. Here, we generated and analyzed the genome sequences from 52 hMPV-positive samples, collected from individuals enrolled in our outpatient clinics/community surveillance studies in Kilifi, Coastal Kenya, between 2016-2017 and 2021-2024. Using the Oxford Nanopore Technologies (ONT) sequencing approach, we recovered hMPV sequences belonging to both known viral lineages, hMPV-A and hMPV-B, which were further classified into three distinct genetic sub-lineages (A2c, B1 and B2b). Over the years, the predominant hMPV sub-lineage in the region changed from B2b in 2016, to B1 in 2021, and then to A2c in 2023. Notably, for the first time in Kenya, we detected a sequence belonging to a newly characterized variant of hMPV, A2c-111nt-dup, that has a 111-nucleotide duplication in the G (glycoprotein) gene. Our findings demonstrate the sub-lineage dynamics of hMPV in Coastal Kenya. We recommend continuous monitoring of hMPV sequence characteristics to track its spread and inform the design of future interventions.

## Introduction

Human metapneumovirus (hMPV) was first described in 2001 in the Netherlands during routine investigation of samples from children with acute respiratory tract disease. ^1^ It has henceforth been reported in several countries globally and is estimated to contribute to 5-15% of all respiratory tract infections. ^2, 3^ Its highest disease burden is in under-two-year-olds, manifesting as either upper respiratory tract infection (URTI) or in severe cases, as lower respiratory tract infections (LRTI). ^4– 6^ hMPV infections also occur in adults and can be severe in immunocompromised individuals or patients with lung/heart co-morbidities. ^7^ Coinfection of hMPV with other respiratory viruses such as respiratory syncytial virus (RSV), severe acute respiratory syndrome coronavirus 2 (SARS-CoV-2), parainfluenza virus, influenza (A/B/C) or adenovirus has been reported, ^7– 11^ and can result in severe disease outcomes, including death. ^12^ In Kenya, a low-middle income East Africa country, hMPV accounts for approximately 5% of acute respiratory infections observed in outpatient and inpatient settings, with peak detection between October and March. ^3, 13^

hMPV is a member of *pneumoviridae* family, genus *Metapneumovirus*, and has an estimated genome size of 13.3kb. ^14^ Its genome is a negative-sense, single-stranded RNA molecule and comprises of 8 open reading frames (ORFs) that encode 9 viral proteins. Three of these proteins i.e., fusion (F), attachment (G), and small hydrophobic (SH) constitute the viral envelope and play a role in attachment (G) to and virion fusion with host cells (F). The function of the short hydrophobic (SH) protein is not well known while the F protein is reported to be the main immunogenic hMPV protein ^1, 15– 17^ and is more conserved than the G protein. ^3, 18, 19^ The nucleocapsid consists of viral RNA associated with the N (nucleoprotein), P (phosphoprotein), and L (large polymerase) proteins, forming a complex, key for genome replication and transcription. The hMPV genome also encodes the Matrix (M), M2-1 and M2-2 proteins, which regulate virus assembly, RNA transcription and replication. ^14, 20^

hMPV is subdivided into two main antigenic lineages (A and B) which are further divided into the A1, A2a, A2b, A2c, B1, and B2 sub-lineages, based on sequence diversity. ^1, 21– 23^ However, an alternative lineage nomenclature has been recently proposed and comprises of sub-genotypes A1, A2, A2.1, A2.2, A2.2.1, A2.2.2, B1 and B2. ^24^ Further, two uniquely diverse A2 sub-lineage strains have been reported, one with a 180-nucleotide duplication, first detected during the 2015/2016 season in Spain ^21^ and another with 111-nucleotide duplication in the G gene, ^25^ first identified in 2017 in Japan, although the clinical significance of these duplications are yet to be established. Although the two duplications were initially thought to have arisen independently, recent analyses suggest that the 111-nucleotide duplication may instead have originated from a duplication–deletion event within the 180-nucleotide variant. ^26, 27^ This hypothesis warrants further investigation. These sub-lineages are distributed globally and show lineage replacement between seasons and geography. ^19, 28, 29^ Additionally, they have been found to co-circulate in the same epidemic period although some are more frequent in some geographic regions relative to others. For instance, in studies from Kenya, Saudi Arabia, and Pakistan, the A1 sub-lineage was not identified in all the analyzed samples, ^3, 18, 30– 32^ and some reports hypothesize its possible extinction. ^10, 24^ Co-infection of these lineages has also been reported. ^10^ However, to date, there has been no evidence of association of particular hMPV lineages/sub-lineages with variations in disease severity. ^15, 32^

Whole genome sequencing (WGS) analysis of hMPV is important in understanding its evolution, transmission, and genetic diversity. ^10, 11, 19, 24, 33^ However, hMPV genomic data are scarce across Africa. ^29^ In coastal Kenya, the most recent complete hMPV sequences were obtained from 4 samples collected more than a decade ago, in 2015. ^34^ In this study, we performed WGS of hMPV-positive samples collected in 2016-2017 and 2021-2023, in Kilifi, Kenya, using the Oxford Nanopore Technologies (ONT) sequencing approach. We observed changes in the dominant sub-lineages from B2b to B1, and then to A2c-wild type. Phylogenetic analysis showed that Kilifi viruses clustered with viruses sampled globally pointing to multiple introductions (importations) of the virus and varied transmission patterns. These data bridge the gap in hMPV genomics in Kenya, providing insights into the recently circulating lineages and the hMPV genomic diversity trends in coastal Kenya.

## Materials and methods

In this section, brand names and manufacturers are included only where necessary to ensure reproducibility, as results may vary between platforms, reagents, or analytical tools.

### Study samples

**Figure 1.**
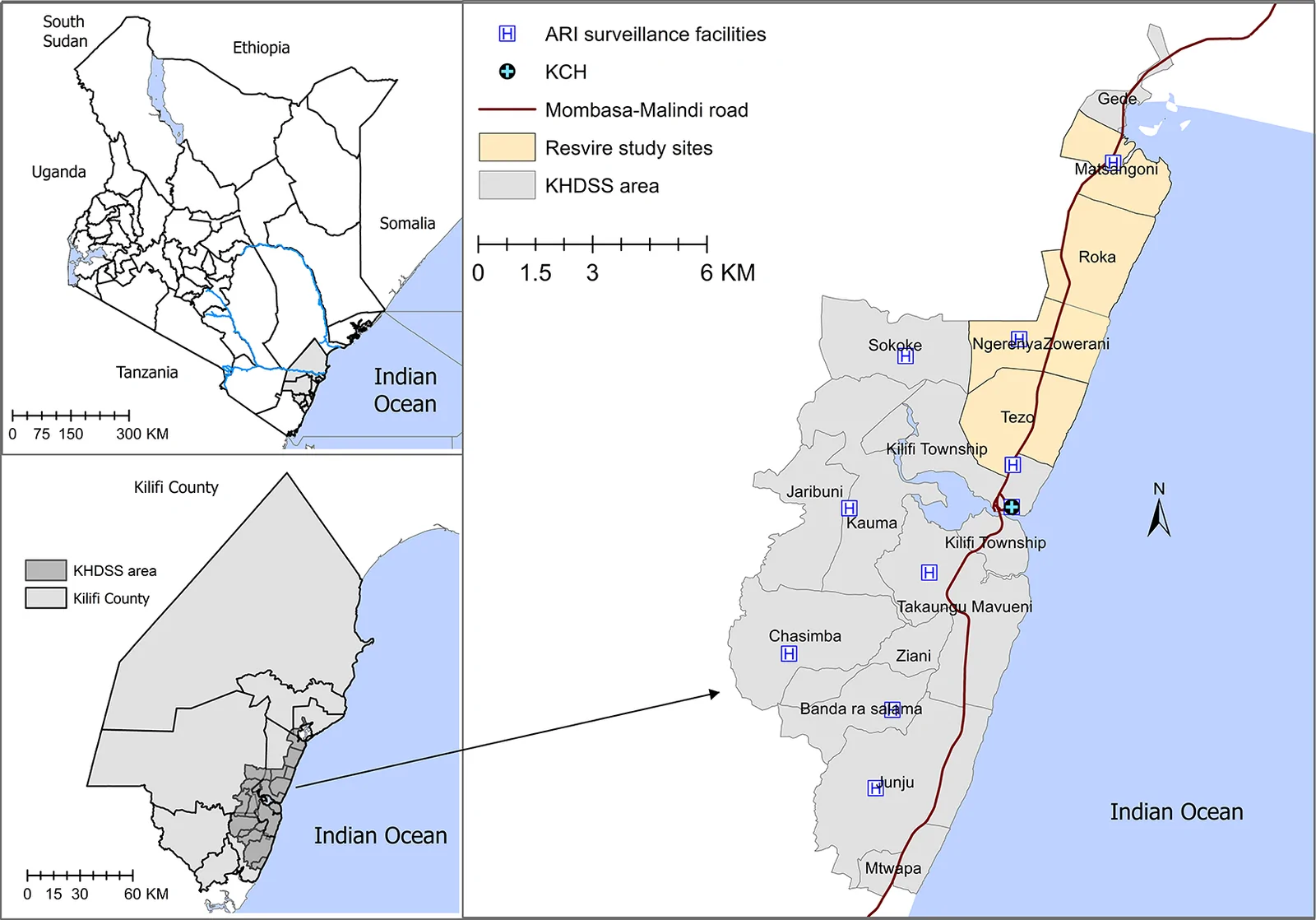
A map of the study area showing Kenya, Kilifi County and Kilifi Health and demographic surveillance system (KHDSS) area. Within the KHDSS, the location of enrolling health facilities (HF) and ResViRe study locations are shown. The top left panel shows the location of Kilifi County relative to the map of Kenya.

A total of 192 archived nasopharyngeal/oropharyngeal (NP/OP) samples that were previously classified as positive for hMPV were processed. These included 149 samples that were collected within Kilifi County, as part of the outpatient Health Facility (HF) Study, ^9^ between 2015 and 2016 and 2020-2024. The HF study aimed to elucidate the epidemiology of respiratory pathogens in Kilifi, Kenya, and was conducted in two phases. The first phase of the study, conducted between December 2015 and 2017, involved nine health facilities (outpatient departments): Chasimba, Jaribuni, Junju, Mtondia, Mavueni, Pingilikani, Ngerenya, Sokoke, and Matsangoni. The second phase, initiated in December 2020 and currently ongoing, includes five facilities: Kilifi County Hospital, Matsangoni, Mavueni, Mtondia, and Pingilikani. In brief, the samples were collected from consenting participants, presenting symptoms of acute respiratory tract infection (ARI), and seeking care from one of the target health facilities, within the Kilifi Health and Demographic Surveillance System (KHDSS). The KHDSS ( Figure 1), established in 2000, is situated in Kilifi County and spans approximately 891 km ^2^, extending about 35 km along Kenya’s coastline. It was set up to assess the burden of childhood diseases, evaluate community-based interventions, and provide a structured sampling framework for epidemiological research. ^35^ As of 2022, the KHDSS population was estimated at around 300,000 individuals. ^36^ Ethical approval was granted by the Scientific and Ethics Review Unit (SERU) of the Kenya Medical Research Institute (SERU#3103) in September 2015.

The other 43 hMPV positive samples were collected as part of respiratory virus reinfections (ResViRe) study (SERU#4724, July 2023) (37 participants. 6 individuals were sampled twice, at different timepoints), which explores the rates, determinants and transmissibility of respiratory virus reinfections within households in Kilifi, Kenya. ^37^ The ResViRe study began in August 2023 and aimed to recruit approximately 450 participants from 65 homesteads. Home visits are conducted one to two times weekly to collect nasopharyngeal and oropharyngeal (NP/OP) swab specimens, irrespective of symptom status. The specimens are then tested for a range of respiratory viral pathogens, including hMPV using a multiplex quantitative PCR assay. Written informed consent was obtained from all participants, or from parents or legal guardians for individuals under 18 years of age, prior to participation in either the HF or ResVire studies.

### hMPV detection by RT qPCR

For both the HF and ResViRe studies, hMPV was detected using a duplex real-time reverse transcription quantitative PCR (RT qPCR) assay that simultaneously targeted hMPV and rhinovirus. For hMPV detection, primers (Forward: ATGTCTCTTCAAGGGATTCACCT; Reverse: AMAGYGTTATTTCTTGTTGCAATGATGA) and a FAM-labeled probe (FAM-CATGCTATATTAAAAGAGTCTCARTAC-BHQ1) targeting the nucleocapsid gene were used. ^38^ Each 10 μL reaction mixture contained 2 μL of RNA, 5 μL of 2× QuantiFast Mix (QIAGEN, Hilden, Germany), 0.2 μL of 50× ROX reference dye, 1 μL of primer-probe mix, 0.1 μL of QuantiFast reverse transcriptase (QIAGEN, Hilden, Germany), and 1.7 μL of nuclease-free water. Thermocycling was performed under the following conditions: 50°C for 20 minutes (reverse transcription), 95°C for 5 minutes (initial denaturation), followed by 40 cycles of 95°C for 15 seconds and 60°C for 30 seconds. Reactions were run on ABI 7500, QuantStudio™ 5, or QuantStudio™ 7 Real-Time PCR Systems (Applied Biosystems). A sample was considered hMPV-positive if the cycle threshold (Ct) value was <35.0 and run positive and negative control results were valid.

### RNA extraction, cDNA synthesis and multiplex PCR

RNA was extracted from 140 μl of hMPV-positive NP/OP samples using the QIAamp Viral RNA Mini Kit (QIAGEN, Hilden, Germany), following the manufactures’ instructions. The extracted RNA was used for cDNA synthesis using the LunaScript ^®^ RT Supermix (New England Biolabs) which utilizes a random priming approach for synthesis. A 10μl cDNA synthesis reaction consisted of 8μl RNA template and 2 μl LunaScript ^®^ RT Supermix. The reaction was incubated in a thermocycler at 25°C for 2 minutes, 55°C for 10 minutes and 95°C for 1 minute. The resulting cDNA was taken forward for second-strand DNA synthesis and multiplex polymerase chain reaction (PCR) using the Q5 ^®^ Hot Start High-Fidelity 2X Master Mix (New England Biolabs), and an 18-amplicon, overlapping primer set designed to span the entire hMPV genome. ^2^ These amplicons were generated in two pools as described previously with a target size of 1000 bp per amplicon. An additional primer set, constituting the third pool, ^39^ was included to fill up a consistent ~4000 bp gap identified in our preliminary sequence data. Each PCR reaction had a total volume of 12.5 μl, consisting of 6.3 μl Q5 ^®^ Hot Start High-Fidelity 2X Master Mix, 1.9 μl primer pool, 3 μl nuclease free water and 1.3 μl cDNA template. The following thermocycling parameters were used for PCR: 1 cycle of 98°C-30 seconds, 38 cycles of 98°C-30 seconds, 50°C 30-seconds and 72°C-2 minutes, and a final extension at 72°C for 10 minutes. Post-PCR products were visualized using 1.5% agarose gels in the ChemiDoc™ XRS+ imaging system (Biorad).

### Post PCR-cleanup, library preparation and sequencing

The PCR products for each sample with visible gel bands across all pools were pooled into one tube and cleaned using Agentcourt AMPure XP beads (Beckman and Colter) at a 1:1, sample-to-bead ratio, then quantified using the Qubit™ 1X dsDNA High Sensitivity Kit (Invitrogen) on the Qubit 4 fluorometer (Invitrogen). All library preparation steps were conducted following the Ligation sequencing amplicons - Native Barcoding Kit 96 V14 (SQK-NBD114.96) protocol (Oxford Nanopore Technologies). End repair was done using the NEBNext ^®^ Ultra™ II End Repair/dA-Tailing Module (New England Biolabs) while subsequent library preparation steps were conducted using the Native Barcoding Kit 96 V14 (ONT), and the NEBNext ^®^ Quick Ligation Module (New England Biolabs). Sequencing was performed on the GridION device (ONT) using the R10.4.1 flow cell, with high accuracy base-calling, for 72 hours.

### Whole-genome assembly

Consensus sequences were generated from individual sample data using a customized in-house bioinformatics pipeline, Viralphyl v0.11.2 ( https://doi.org/10.5281/zenodo.17055772). Amplicon reads were aligned to a panel of hMPV reference genomes downloaded from GenBank (accession numbers: KU821121.1, OL794442.1, KJ627387.1, OL794460.1, MN745085.1, MK167039.2, OL794440.1, MZ504964.1 and OL794427.1), representing sub-lineages A1, A2a, A2b, A2c-wt, A2c-dup111, A2c-dup180, B1, B2a and B2b, respectively. Briefly, raw FAST5 files were initially converted to FASTQ, followed by demultiplexing using ONT Dorado v7.3.9 with default settings. Subsequent filtering steps excluded reads shorter than 500 base pairs and those with a Phred score of less than 9. Barcode-specific reads were then mapped to multiple hMPV reference genomes using the corresponding primer scheme ( https://github.com/kwtrp-peo/viralphyl/tree/main/primerschemes/hmpv/V1.3.0). The most appropriate reference sequence was selected for each sample to optimize mapping quality. Consensus sequences were further refined using clair3 v1.1.1 ( https://github.com/HKU-BAL/Clair3). Genomic positions with low coverage (read depth <20) were masked with the ambiguous base N. The sequences have been deposited on GenBank and their accession numbers are provided in Table 1.

**Table 1.** hMPV sequences from this study with associated GenBank accession numbers.

Isolate ID	Accession	Study	Genome length	Sub-lineage (phylogenetics)	Nextclade Sub-lineage	Age (years)	Collection date
Kilifi/hMPV/A2/HF147079/03-10-2016	PX279637	HF	13,289	A2c-wt	A2.2.2	2	2016-10-03
Kilifi/hMPV/A2/HF166986/24-08-2016	PX279638	HF	13,290	A2c-wt	A2.2.2	0	2016-08-24
Kilifi/hMPV/A2/HF187198/23-11-2016	PX279639	HF	13,290	A2c-wt	A2.2.2	1	2016-11-23
Kilifi/hMPV/A2/HF196858/16-05-2016	PX279640	HF	13,296	A2c-wt	A2.2.2	3	2016-05-16
Kilifi/hMPV/A2/HF197025/02-08-2016	PX279641	HF	13,290	A2c-wt	A2.2.2	0	2016-08-02
Kilifi/hMPV/A2/HF206578/14-12-2015	PX279642	HF	11,428	A2c-wt	A2.2.2	3	2015-12-14
Kilifi/hMPV/A2/HF207288/02-03-2017	PX279643	HF	12,343	A2c-wt	A2.2.2	5	2017-03-02
Kilifi/hMPV/A2/HF227105/17-10-2016	PX279644	HF	13,292	A2c-wt	A2.2.2	4	2016-10-17
Kilifi/hMPV/A2/HF227108/24-10-2016	PX279645	HF	13,292	A2c-wt	A2.2.2	2	2016-10-24
Kilifi/hMPV/A2/HF246591/19-02-2021	PX279635	HF	13,408	A2c-dup111	A2.2.2	0	2021-02-19
Kilifi/hMPV/A2/RV105723/06-11-2023	PX279646	ResViRe	12,195	A2c-wt	A2.2.2	0	2023-11-06
Kilifi/hMPV/A2/RV177584/08-11-2023	PX279647	ResViRe	13,289	A2c-wt	A2.2.2	10	2023-11-08
Kilifi/hMPV/A2/RV177586/15-11-2023	PX279648	ResViRe	12,892	A2c-wt	A2.2.2	5	2023-11-15
Kilifi/hMPV/A2/RV195685/27-09-2023	PX279649	ResViRe	12,316	A2c-wt	A2.2.2	6	2023-09-27
Kilifi/hMPV/A2/RV64726/17-11-2023	PX279650	ResViRe	11,919	A2c-wt	A2.2.2	0	2023-11-17
Kilifi/hMPV/A2/RV65542/18-10-2023	PX279651	ResViRe	13,289	A2c-wt	A2.2.2	0	2023-10-18
Kilifi/hMPV/A2/RV65563/12-10-2023	PX279652	ResViRe	13,289	A2c-wt	A2.2.2	5	2023-10-12
Kilifi/hMPV/A2/RV65572/06-11-2023	PX279653	ResViRe	11,919	A2c-wt	A2.2.2	0	2023-11-06
Kilifi/hMPV/A2/RV65573/06-11-2023	PX279654	ResViRe	12,316	A2c-wt	A2.2.2	59	2023-11-06
Kilifi/hMPV/A2/RV65574/06-11-2023	PX279655	ResViRe	13,289	A2c-wt	A2.2.2	3	2023-11-06
Kilifi/hMPV/A2/RV77106/27-09-2023	PX279656	ResViRe	12,316	A2c-wt	A2.2.2	0	2023-09-27
Kilifi/hMPV/A2/RV77689/31-10-2023	PX279657	ResViRe	12,892	A2c-wt	A2.2.2	2	2023-10-31
Kilifi/hMPV/A2/RV848202/04-12-2023	PX279658	ResViRe	13,289	A2c-wt	A2.2.2	0	2023-12-04
Kilifi/hMPV/A2/RV848203/07-12-2023	PX279659	ResViRe	11,914	A2c-wt	A2.2.2	0	2023-12-07
Kilifi/hMPV/A2/RV848204/04-12-2023	PX279660	ResViRe	12,316	A2c-wt	A2.2.2	5	2023-12-04
Kilifi/hMPV/A2/RV848205/04-12-2023	PX279661	ResViRe	12,710	A2c-wt	A2.2.2	5	2023-12-04
Kilifi/hMPV/A2/RV866052/01-11-2023	PX279662	ResViRe	12,316	A2c-wt	A2.2.2	2	2023-11-01
Kilifi/hMPV/A2/RV866053/08-11-2023	PX279663	ResViRe	12,316	A2c-wt	A2.2.2	0	2023-11-08
Kilifi/hMPV/B1/HF246854/25-08-2021	PX279664	HF	13,249	B1	B1	22	2021-08-25
Kilifi/hMPV/B1/HF256712/13-04-2021	PX279665	HF	13,248	B1	B1	0	2021-04-13
Kilifi/hMPV/B1/HF256880/19-07-2021	PX279666	HF	12,887	B1	B1	6	2021-07-19
Kilifi/hMPV/B1/HF256967/09-09-2021	PX279667	HF	12,883	B1	B1	5	2021-09-09
Kilifi/hMPV/B1/HF266654/17-03-2021	PX279668	HF	12,885	B1	B1	4	2021-03-17
Kilifi/hMPV/B1/HF266958/06-09-2021	PX279669	HF	12,125	B1	B1	15	2021-09-06
Kilifi/hMPV/B1/HF267640/12-09-2022	PX279670	HF	10,662	B1	B1	2	2022-09-12
Kilifi/hMPV/B1/HF276622/15-02-2021	PX279671	HF	12,884	B1	B1	3	2021-02-15
Kilifi/hMPV/B1/HF276624/15-02-2021	PX279672	HF	12,884	B1	B1	5	2021-02-15
Kilifi/hMPV/B1/HF276653/01-03-2021	PX279673	HF	12,885	B1	B1	0	2021-03-01
Kilifi/hMPV/B1/HF276660/03-03-2021	PX279674	HF	12,885	B1	B1	34	2021-03-03
Kilifi/hMPV/B1/HF276680/16-03-2021	PX279675	HF	12,885	B1	B1	15	2021-03-16
Kilifi/hMPV/B1/HF286808/24-05-2021	PX279676	HF	11,422	B1	B1	1	2021-05-24
Kilifi/hMPV/B2/HF157146/14-11-2016	PX279678	HF	12,118	B2b	B2	5	2016-11-14
Kilifi/hMPV/B2/HF167123/24-10-2016	PX279679	HF	12,883	B2b	B2	25	2016-10-24
Kilifi/hMPV/B2/HF167138/03-11-2016	PX279680	HF	12,876	B2b	B2	22	2016-11-03
Kilifi/hMPV/B2/HF187153/31-10-2016	PX279681	HF	13,241	B2b	B2	1	2016-10-31
Kilifi/hMPV/B2/HF197180/17-10-2016	PX279682	HF	12,119	B2b	B2	5	2016-10-17
Kilifi/hMPV/B2/HF206885/29-06-2016	PX279683	HF	11,364	B2b	B2	1	2016-06-29
Kilifi/hMPV/B2/HF207043/16-09-2016	PX279684	HF	11,363	B2b	B2	2	2016-09-16
Kilifi/hMPV/B2/HF207044/16-09-2016	PX279685	HF	12,877	B2b	B2	44	2016-09-16
Kilifi/hMPV/B2/HF226901/15-07-2016	PX279686	HF	13,241	B2b	B2	1	2016-07-15
Kilifi/hMPV/B2/HF227280/01-03-2017	PX279677	HF	12,859	B2b	B2	2	2017-03-01
Kilifi/hMPV/B2/HF286989/07-10-2021	PX279688	HF	13,243	B2b	B2	4	2021-10-07

### hMPV genotyping and phylogenetic analysis

To characterize the genetic diversity of hMPV, we retrieved annotated reference sequences with known linages from GenBank using the NCBI dataset tool, ^40^ including 136 whole-genome, 136 F gene, and 270 G gene sequences. Additionally, global whole-genome sequences with complete metadata (collection date and location) were downloaded from GenBank (via the NCBI Virus portal) on January 30, 2025. These comprised 325 lineage A and 200 lineage B sequences. The global dataset was grouped by region (Africa, Asia, Oceania, North America, South America) and subsampled by country, region, and date using augur v29.1.0, ^41^ resulting in 177 lineage A and 117 lineage B sequences.

From our study, we generated 52 hMPV genomes: 27 lineage A and 25 lineage B. The G and F genes were extracted from these assemblies using BLAST, ^42^ creating three datasets: whole genome, F gene, and G gene. Each dataset was aligned with the global reference and subsampled sequences using MAFFT v3.23. ^43^ Phylogenetic trees were constructed using the maximum likelihood (ML) method in IQ-TREE v2.3, ^44^ and the best-fit substitution models were determined using ModelFinder. The TIM2+F+I+G4 model was used for the F and G gene datasets, while GTR+F+I+G4 was used for whole-genome sequences. Statistical support for the phylogenies was evaluated using 1,000 bootstrap replicates. In addition, genotyping was performed using Nextclade v3.18.1 with whole-genome sequences as input.

Time-resolved phylogenetic trees for both genotypes were inferred using TreeTime v0.11.4 ^45^ with sampling dates used to estimate a molecular clock and to reconstruct divergence times. These analyses were performed on the whole-genome alignments with the same subsampled global dataset, enabling visualization of the temporal signal and estimating the timing of lineage divergence.

### Statistical analysis

Continuous variables were summarized using mean (standard deviation) or median (interquartile [Q1, Q3]) as appropriate. Categorical variables were presented as counts (%) and stratified by surveillance period ( Table 2) and age categories ( Table 4). Differences in continuous variables across groups were assessed using the Kruskal–Wallis test. When significant differences were identified (p < 0.05), post-hoc pairwise comparisons were performed using Dunn’s test with Bonferroni correction for multiple comparisons. For categorical variables, Pearson Chi-Square test was used when expected cell counts were >5; otherwise, Fisher’s Exact test was applied. Statistical analysis was conducted using R version 4.3.2 and a two-sided p-value of <0.05 was considered significant.

**Table 2.** Baseline demographic characteristics of hMPV-positive individuals from Kilifi.

Characteristic	HF (2016–2017), N = 95 ^1^	HF (2021–2022), N = 54 ^1^	ResViRe (2023–2024), N = 37 ^1^	p-value ^2^
Ct values				<0.001
Mean (±SD)	29.9 (± 3.2)	27.1 (± 3.1)	27.6 (± 3.6)	
Median (Q1, Q3)	29.9 (27.4, 32.7)	26.7 (25.0, 29.1)	28.0 (25.3, 30.7)	
Age (years)				<0.001
Mean (±SD)	4 (± 8)	10 (± 11)	9 (± 13)	
Median (Q1, Q3)	2 (1, 4)	6 (3, 14)	5 (1, 10)	
Age Group (years)				<0.001
1 year	19 (20%)	4 (7.4%)	9 (24%)	
1-2 years	42 (44%)	7 (13%)	5 (14%)	
3-5 years	22 (23%)	16 (30%)	8 (22%)	
6-14 years	4 (4.2%)	14 (26%)	9 (24%)	
> 15 years	8 (8.4%)	13 (24%)	6 (16%)	
Sex				0.603
Female	52 (55%)	34 (63%)	22 (59%)	
Male	43 (45%)	20 (37%)	15 (41%)	
Facility				
Chasimba	13 (14%)	0 (0%)	0 (0%)	
Jaribuni	6 (6.3%)	0 (0%)	0 (0%)	
Junju	9 (9.5%)	0 (0%)	0 (0%)	
Kilifi County Hospital	0 (0%)	5 (9.3%)	0 (0%)	
Matsangoni	12 (13%)	13 (24%)	15 (41%)	
Mavueni	7 (7.4%)	16 (30%)	0 (0%)	
Mtondia	13 (14%)	11 (20%)	0 (0%)	
Ngerenya	8 (8.4%)	0 (0%)	5 (14%)	

Data are presented as mean (SD), median (IQR: Q1, Q3), or as percentages (n/N). Percentages are based on the total number of observations within each column for the study period. ResViRe study samples were collected from households across various locations in Kilifi, rather than from health facilities. HF - Health Facility study.

^1^ n (%).

^2^ Kruskal-Wallis rank sum test; Pearson’s Chi-squares; Bold P-values represent significance at 5% alpha level.

## Results

### Baseline characteristics and transmission patterns of hMPV

**Figure 2.**
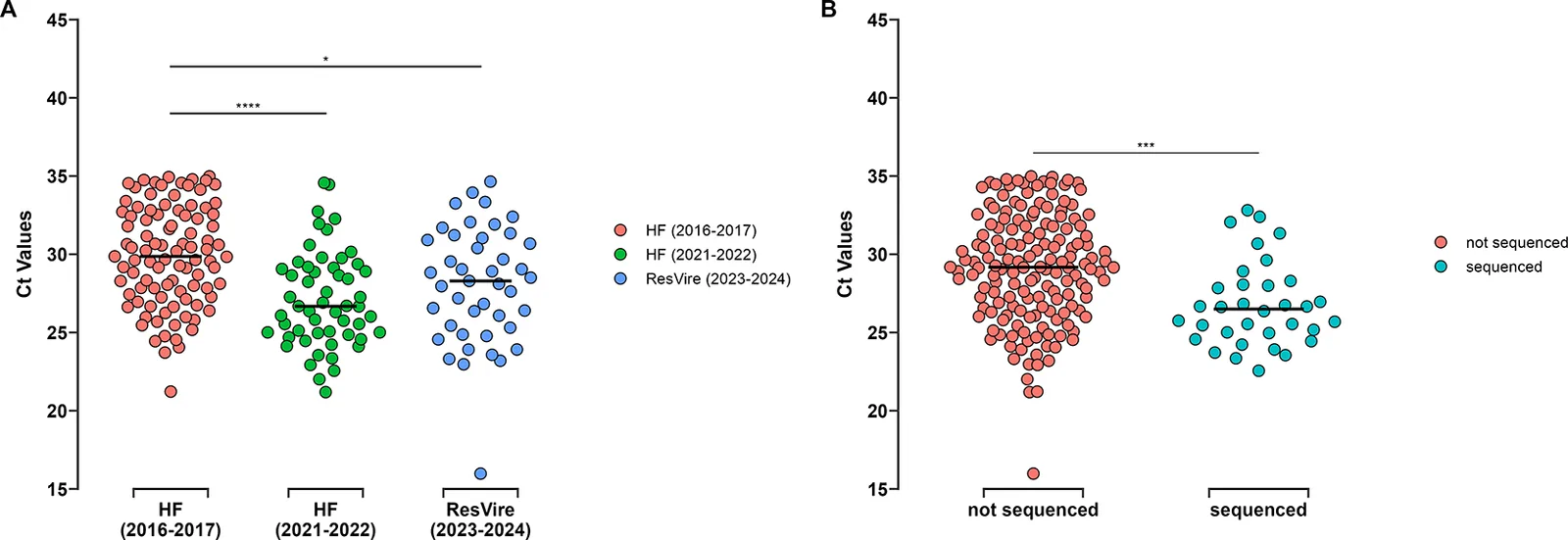
(A) Ct values across the studies. Values were obtained from qPCR testing of nasopharyngeal/oropharyngeal samples collected in health facilities (HF) and households (ResVire) in Kilifi. hMPV positivity was based on a Ct cut-off of 35. The median Ct values were compared using the Kruskal-Wallis test. * Indicates p = 0.011, while **** shows that p < 0.0005. **(B)** Ct values according to sequencing status. Samples were categorized as “sequenced” or “not sequenced” based on whether sequencing was successful. The median Ct values were compared using the Wilcoxon rank-sum test where *** denotes p < 0.001, indicating a statistically significant difference between the groups.

**Figure 3.**
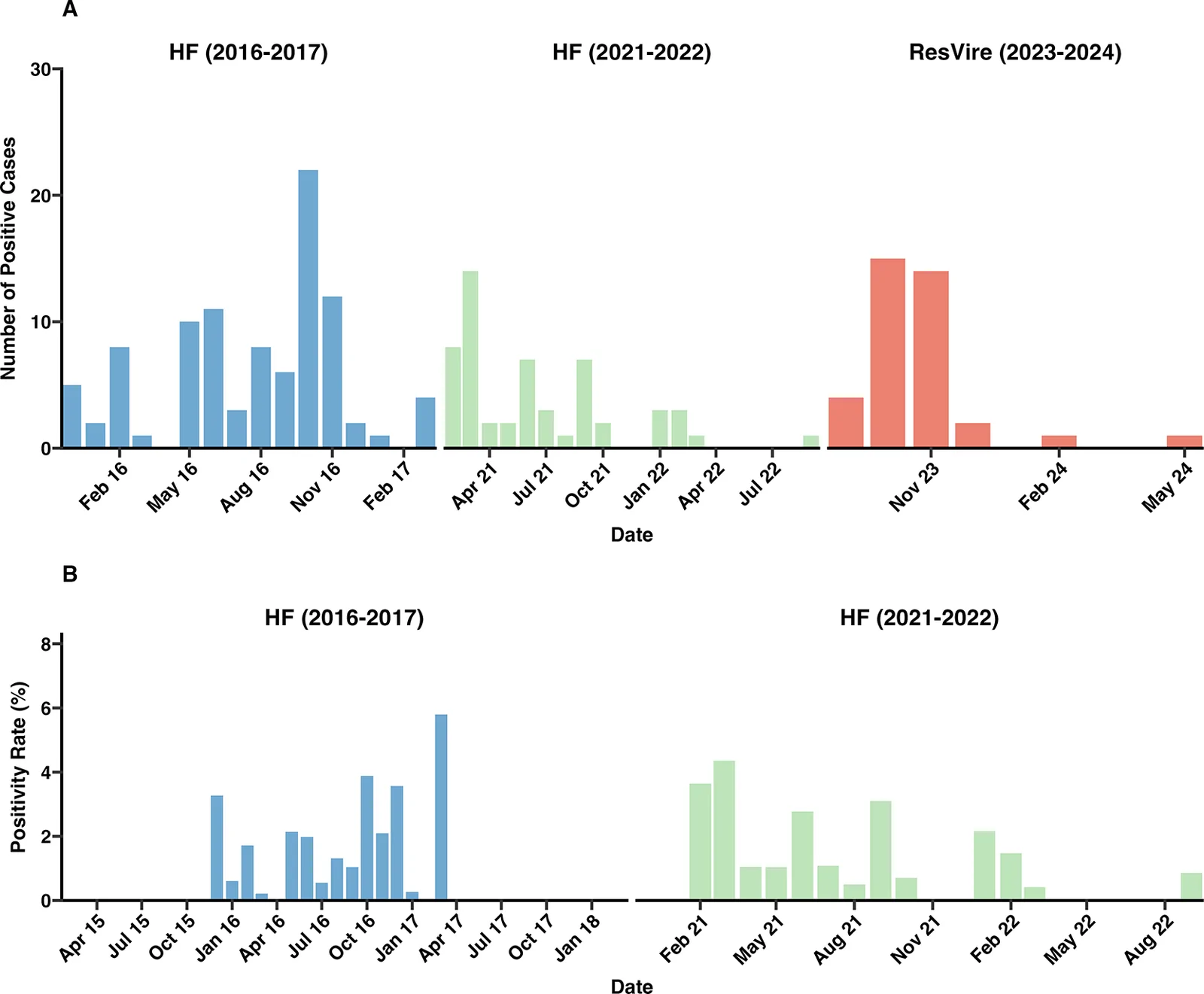
Monthly hMPV positivity and detection rates across three study periods. hMPV was detected by qPCR in nasopharyngeal/oropharyngeal samples collected from health facilities (HF) and households (ResVire) in Kilifi. **(A)** Absolute number of laboratory-confirmed hMPV-positive cases per month, stratified by study period, with bar shading used to distinguish the study years. **(B)** Monthly hMPV positivity rate (%), calculated as (number of positive tests divided by the total tests performed) × 100, shown for HF 2016–2017 and HF 2021–2022.

The baseline characteristics of the individuals analyzed here are summarized in Table 2. Across all study years (2016-2017 and 2021-2023 for HF, and 2023-2024 for ResViRe), there were more female participants than male - 55%, 63%, and 59% versus 45%, 37%, and 41%, respectively. Children under 5 years accounted for most hMPV positive cases (72%). The median age of participants differed significantly across study periods (p < 0.001), with the highest median age observed among participants in the 2021–2022 HF study. Participants in 2016–2017 were younger (median age: 2, IQR: 1-4) than those in later study periods. No difference in age was observed between 2021–2022 and 2023–2024 ( Table 3). The median cycle threshold (Ct) value among positive samples also differed significantly across study periods, with the highest median Ct recorded between 2016 and 2017. Additionally, samples that were successfully sequenced had significantly (p < 0.001) lower Ct values than those that were not ( Figure 2). It is noteworthy that whereas the HF study recruits patients with ARI symptoms, participants in the ResViRe study provided a sample regardless of symptom status. The most frequently recorded clinical symptoms among symptomatic cases included cough (82.4%), fever (41.5%), and nasal discharge (22.6%), predominantly affecting children under five years. A breakdown of baseline characteristics by participants age is provided in Table 4. hMPV displayed a seasonal pattern, with the most cases recorded between August and December ( Figure 3), consistent with earlier findings. ^3^

**Table 3.** Dunn’s test post-hoc pairwise comparisons for Ct values and age.

Comparison	Ct values (Z)	Ct values (adjusted p)	Age (Z)	Age (adjusted p)
2016-2017 vs 2021-2022	4.870	<0.001	-5.182	<0.001
2016-2017 vs 2023-2024	3.206	0.002	-2.637	0.013
2021-2022 vs 2023-2024	-0.978	0.492	1.744	0.122

Following significant Kruskal-Wallis tests (p < 0.001 for both Ct values and age), Dunn’s tests with Bonferroni correction were performed to identify which specific time periods differed. Adjusted p-values < 0.05 indicate statistical significance. The table presents the test statistic (Z) and Bonferroni-adjusted p-value for each pairwise comparison.

**Table 4.** Characteristics of included individuals from the Health Facility and ResViRe studies, stratified by age < 15 years.

Characteristic	1 year, (n = 32)	1-2 years, (n = 54)	3-5 years, (n = 46)	6-14 years, (n = 27)	P-value
**Ct values**					0.462
Mean (± SD)	28.9 (± 3.4)	28.3 (± 3.6)	29.2 (± 3.3)	29.4 (± 2.9)	
Median (Range)	29.1 (22.6, 34.9)	28.1 (21.2, 34.7)	29.4 (23.2, 35.0)	28.6 (24.7, 34.6)	
**Sex**					0.395
Female	13 (41%)	30 (56%)	27 (59%)	16 (59%)	
Male	19 (59%)	24 (44%)	19 (41%)	11 (41%)	
**Temperature**					0.967
Mean (± SD)	35.83 (± 0.53)	35.90 (± 0.44)	36.15 (± 0.90)	35.87 (± 0.58)	
Median (Range)	36.00 (35.10, 36.50)	36.10 (35.30, 36.40)	36.05 (35.00, 38.10)	36.00 (35.00, 36.60)	
**Health Facility/Location**					
Chasimba	3 (9.4%)	8 (15%)	2 (4.3%)	0 (0%)	
Jaribuni	0 (0%)	5 (9.3%)	1 (2.2%)	0 (0%)	
Junju	3 (9.4%)	1 (1.9%)	2 (4.3%)	0 (0%)	
Kilifi County Hospital	2 (6.3%)	0 (0%)	1 (2.2%)	0 (0%)	
Matsangoni	8 (25%)	10 (19%)	6 (13%)	11 (41%)	
Mavueni	3 (9.4%)	6 (11%)	5 (11%)	4 (15%)	
Mtondia	3 (9.4%)	3 (5.6%)	10 (22%)	5 (19%)	
Ngerenya	2 (6.3%)	5 (9.3%)	3 (6.5%)	1 (3.7%)	
Pingilikani	1 (3.1%)	2 (3.7%)	4 (8.7%)	1 (3.7%)	
Roka	3 (9.4%)	4 (7.4%)	5 (11%)	4 (15%)	
Sokoke	4 (13%)	10 (19%)	7 (15%)	1 (3.7%)	
**Cough**	24 (75%)	50 (93%)	39 (85%)	18 (67%)	0.018
**Headache**	0 (0%)	0 (0%)	2 (8.3%)	1 (4.3%)	0.769
**Running Nose**	1 (11%)	2 (40%)	0 (0%)	0 (0%)	0.110
**Sore Throat**	2 (6.3%)	5 (9.3%)	6 (13%)	3 (11%)	0.838
**Wheezing**	1 (11%)	0 (0%)	0 (0%)	0 (0%)	1.00
**Vomiting**	1 (7.7%)	0 (0%)	2 (8.3%)	0 (0%)	0.498
**Difficulty in Breathing**	2 (15%)	1 (8.3%)	3 (13%)	0 (0%)	0.245
**Fever**	12 (43%)	33 (70%)	18 (60%)	3 (23%)	0.008
**Nasal Discharge**	4 (100%)	4 (57%)	16 (100%)	12 (86%)	0.026
**Nasal Flaring**	0 (0%)	0 (0%)	3 (19%)	1 (7.1%)	0.675
**Crackles**	2 (8.7%)	1 (2.0%)	1 (2.6%)	0 (0%)	0.435
**Wheezes**	1 (25%)	1 (14%)	0 (0%)	0 (0%)	0.067
**Unable to Feed**	1 (25%)	0 (0%)	0 (0%)	0 (0%)	0.098
**Cyanosis**	1 (4.3%)	0 (0%)	0 (0%)	0 (0%)	0.320
**Body Malaise**	0 (0%)	1 (14%)	2 (13%)	3 (21%)	0.921
**Abdominal Pains**	0 (0%)	0 (0%)	0 (0%)	1 (7.1%)	0.610
**Chest Pain**	0 (0%)	0 (0%)	0 (0%)	2 (14%)	0.324
**Dizziness**	0 (0%)	0 (0%)	0 (0%)	1 (7.1%)	0.610

Data are presented as either mean (SD), median (range, min-max), percentage and (n/N). Percentages are expressed as counts and the denominator is column totals stratified by age group. ResViRe study samples were collected from households in different locations.

### Lineage assignment and phylogenetics

All the 192 hMPV-positive samples obtained from both the ResViRe (n = 43) and HF studies (n = 149) were targeted for whole-genome sequencing. Of these, 72 were dropped post-PCR during quality control (lacked visible gel bands in at least 1 of the 3 amplification pools or had lower than 18 ng/μl Qubit concentration, our minimum quantity required to proceed to library preparation). A further 68 samples were dropped post-sequencing due to low coverage breadth (>30% Ns). Complete or near complete genomes (defined as genome coverage >70%) were obtained from 52 samples, with a medium genome size of 12,844 bp (range: 10,662 – 13,408 bp) and an average read depth of 23. Of these, two sequences originated from a single participant who was sampled at different time points in accordance with the ResViRe protocol. These 52 sequences were taken forward for further downstream analyses, including lineage assignment and phylogenetic analysis.

**Figure 4.**
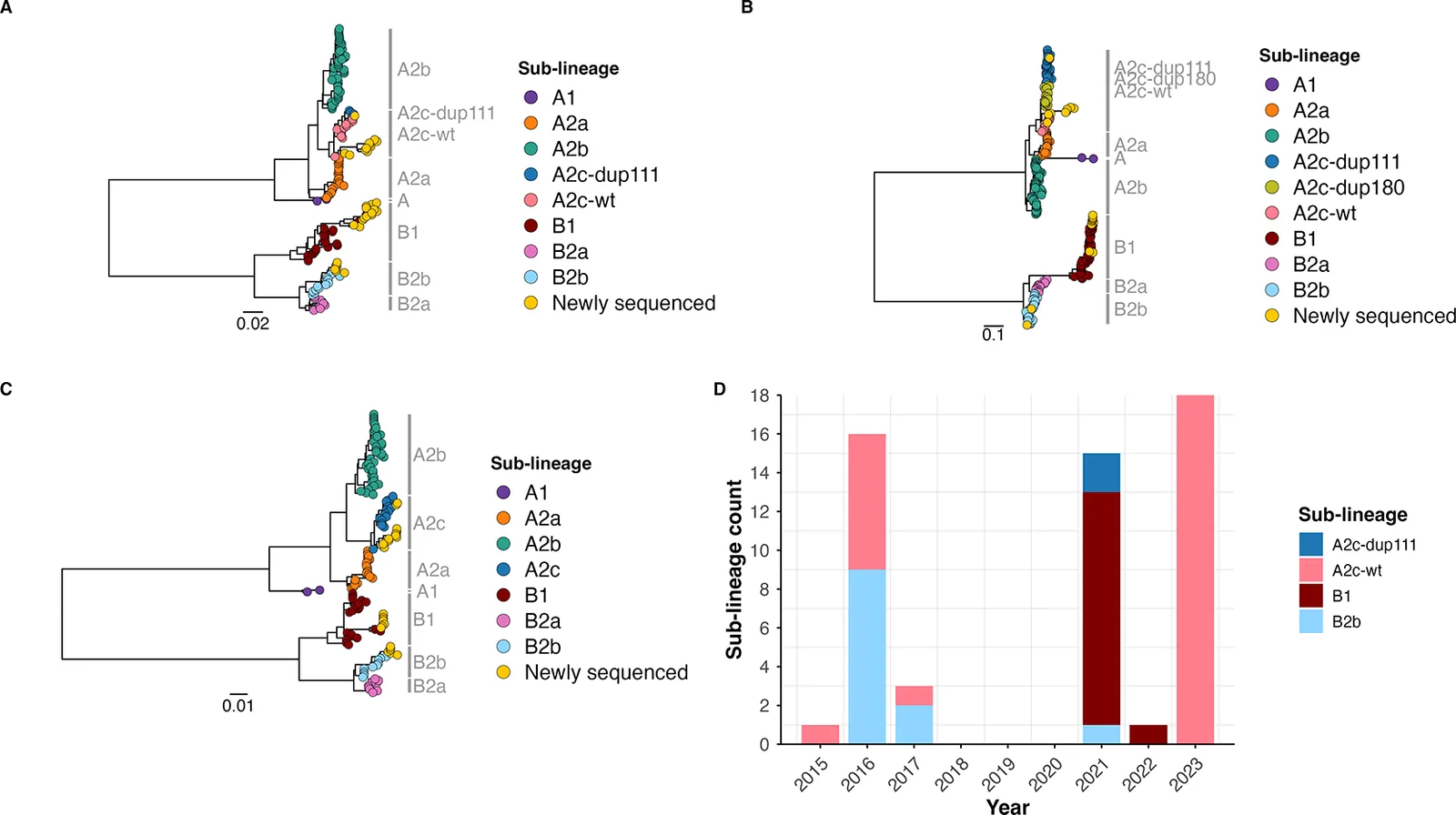
Phylogenetic clustering of the 52 hMPV strains sequenced in this study. Lineage assignment was performed using both whole genome and complete F and G gene sequences. Maximum likelihood (ML) phylogenetic trees were constructed using ( **A**) whole genome, ( **B**) G gene, and ( **C**) F gene sequences. The TIM2+F+I+G4 model was used for the F and G sequences while the GTR+F+I+G4 model was employed for whole genome sequences, both with 1000 bootstrap replicates for statistical support. ( **D**) Temporal distribution of identified hMPV sub-lineages from 2015 to 2023. Bars represent sub-lineage counts. Wt-wild type. Dup111-G gene with 111-nucleotide duplication. Dup180-G gene with 180 nucleotide duplication.

Lineage assignment was conducted using two complementary approaches. First, lineage assignment was performed in Nextclade, using whole-genome sequences ( Table 1). Second, phylogenetic clustering was performed by comparing study sequences with 136 reference sequences retrieved from GenBank. For this approach, lineage assignment was conducted separately using whole genome as well as complete G and F gene sequences. All three phylogenetic analyses yielded consistent lineage/sub-lineage assignments, each supported by over 80% bootstrap values ( Figure 4, A-C). Out of the 52 sequences used for lineage assignment, 27 (51.9%) were grouped as sub-lineage A2c-wt (including the two that originated from the same participant), 12 (23.1%) as B2b, and 13 (25%) were classified as B1 ( Table 1). We identified one sample within the A2c group (dated 19 ^th^ February 2021), with the 111-nucleotide duplication in the G-gene. The sample was collected during Phase 2 of the HF study. The sub-lineages A1, A2a and A2b were not identified in any of the sequences obtained from this study. Overall, lineages A and B were observed to alternate in dominance, over the years. For instance, in 2016, the predominant sub-lineage was B2b, but in 2023, A2c dominated ( Figure 4 D). The distribution of lineages/sub-lineages between 2018 and 2020 could not be established as surveillance was disrupted during this period.

### Global placement of Kilifi hMPV sub-lineages

We evaluated the phylogenetic placement of hMPV lineages A and B genomes from this study in comparison to previously published global genomes (n = 287). The global genomes were grouped into six regions and sub-sampled for both hMPV-A (n = 170) and hMPV-B (n = 117) ( Table 5). Many of the sequences for both lineages were from the Netherlands (A: 33/170, 19.4%; B: 30/117, 25.6%), the USA (A: 43/170, 25.3%; B: 52/117, 44.4%), China (A: 34/170, 20.0%; B: 14/117, 12.0%) and Peru (A: 38/170, 22.4%; B: 7/117, 6.0%). Africa was the least represented region, with only two sequences per lineage.

**Table 5.** Regional representation of hMPV genomes included in the global analysis. Genomes generated in this study are grouped as Kilifi, while previously published genomes (n = 287) are categorized by world region and genotype (hMPV-A or hMPV-B).

Region/Lineage	A	B	Total
Africa	2	2	**4**
Asia	36	17	**53**
Europe	39	30	**69**
North America	43	52	**95**
Oceania	11	8	**19**
South America	39	8	**47**
Kilifi	28	24	**52**
**Total**	**197**	**122**	**319**

**Figure 5.**
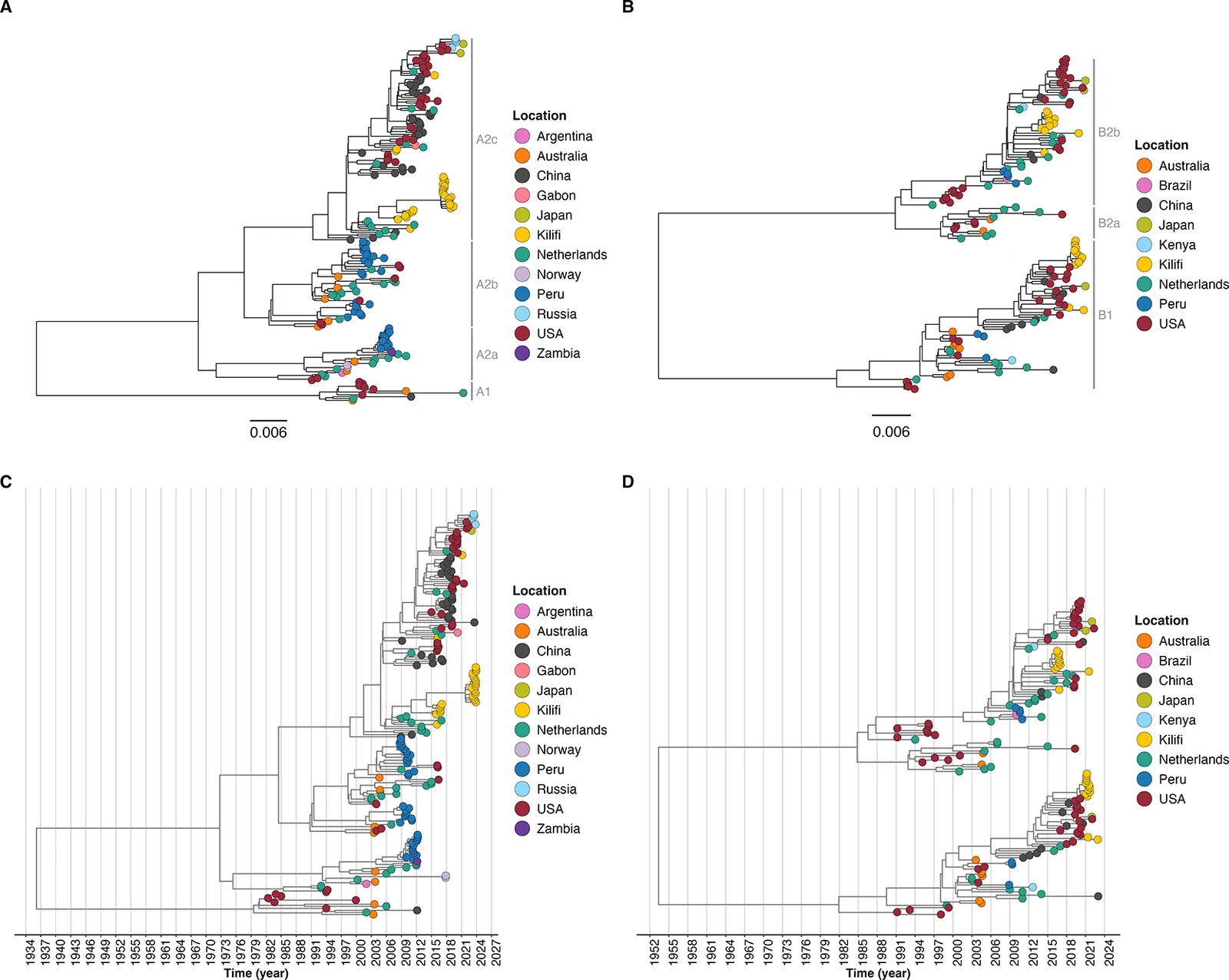
Global phylogenetic placement of hMPV-A and hMPV-B genomes from Kilifi, Kenya. (A-B) Maximum likelihood phylogenetic trees of hMPV lineage A (n = 170) and B (n = 117), inferred using the GTR+F+I+G4 model and 1,000 bootstrap replicates. (C-D) Time-resolved phylogenetic trees for lineages A and B generated with TreeTime, illustrating estimated divergence over time. Tree tips are colored by sampling location (country or region) to show the global distribution of reference sequences. Genomes from this study (Kilifi) are labeled and highlighted in golden yellow to indicate their placement relative to global diversity.

Most hMPV-A sequences from Kilifi clustered closely with those from the Netherlands ( Figure 5) which may reflect the effect of the disproportionately high number of Dutch sequences in the global dataset, reflecting potential sampling bias. Additionally, we identified a monophyletic cluster comprising 24 A2c-wt strains (85.7%, 24/28), all originating from Kilifi, forming a distinct local lineage closely related to strains from the Netherlands, sampled in 2009/2010. This pattern could indicate sustained local circulation in Kenya with limited genetic change over time or may simply reflect gaps and biases in the available genomic data from other regions and periods. The Kilifi A2c-wt cluster also exhibited clear separation between the 2023 sequences and those from 2016/2017 in the phylogenetic tree, consistent with genetic divergence over time within the same lineage. Furthermore, two A2c-wt sequences formed a cluster closely related to 2009 sequences from China, which could be due to under-sampling of this sub-lineage in other regions or years.

The hMPV-B sequences from Kilifi clustered into two distinct local groups. The first consisted of 13 B1 sequences (54.2%, 13/24) from 2021, which formed a monophyletic group with sequences from the USA collected in 2019/2020. The second group included 11 B2b strains (45.8%, 11/24) from 2016, showing close genetic relatedness to 2018 sequences from the Netherlands ( Figure 5B). These observed phylogenetic relationships may reflect shared ancestry or convergent clustering in the context of limited and uneven sampling across regions and years.

## Discussion

We analyzed 52 near-complete hMPV genomes generated using the ONT sequencing platform from participants of local ARI surveillance in Kilifi, coastal Kenya. These data add to the limited hMPV genomic information available for Kenya (n = 15) and indeed for Africa (n = 19), providing an updated view of the recent hMPV molecular epidemiology in the region. These sequences can also be used as references to improve the design of molecular diagnostics and sequencing primers for the optimal genomic surveillance of emerging regional variants.

Our analysis confirmed local circulation of both known hMPV lineages A and B, across the study periods, with a change in the predominant sub-lineage over time. This corroborates previous reports which showed that hMPV A & B lineages co-circulate within seasons, alternating in predominance across seasons. ^2, 10, 32, 34^ No strain belonging the A1 sub-lineage was identified in this study, consistent with previous findings, pointing towards its possible extinction. ^24^

For the first time in Kilifi and indeed Kenya, we report detection in one sample collected in 2021, the A2c sub-lineage with 111-nucleotide duplication in the G-gene. As these duplications were first reported in 2017, ^25^ our findings underscore the importance of ongoing viral genomic surveillance, for the identification of emerging hMPV variants. However, we cannot conclude on the exact year/date the A2c-111nt-dup strains were introduced into Kenya since our surveillance was disrupted between 2018 and 2020. Additional and representative sequence data are needed to better understand the origin and evolution of the duplication variants. A recent study suggests that the 111-nucleotide variant arose from a duplication–deletion event within the existing 180-nucleotide region. ^26, 27^ Further, we did not detect any A2c sequences with the 180-nucleotide duplication in the G Gene, that have recently been reported in other regions. ^10, 24, 29, 46^ This could be due to the small sample size analyzed here.

Genotype analysis using whole genome, G and F gene sequences, yielded consistent results across all the methods. This has been reported elsewhere, ^19, 28^ indicating full genome, F or G gene sequences provide consistent hMPV genotyping results. This may be particularly useful when whole genome sequencing fails due to ether primer mismatches or low virus load but will still allow identification of the infecting lineage and sub-lineage.

Our phylogenetic analysis showed that the sequences from Kilifi clustered with those from other African regions and countries like China, USA, and the Netherlands, as reported previously. ^3, 32, 34^ This pattern may suggest multiple introductions of the virus into Kilifi and a shared most recent common ancestor among these regions. Like other respiratory viruses, hMPV spreads rapidly across regions, largely following patterns of human mobility. However, this genetic closeness could also reflect a bias in sequence availability on GenBank, where most of the deposited sequences originate from the USA, China, and the Netherlands. Given that only a tiny fraction of yearly hMPV infections undergo genome sequencing and genomic surveillance is concentrated in a few countries, conducting a robust phylogeographic analysis that mitigates sampling bias remains challenging at present.

The highest number of hMPV cases were documented in children aged <5 years, a pattern consistent with the known epidemiology of hMPV. ^1, 3, 19, 47^ In addition, the most prevalent clinical symptoms (cough, fever, and nasal discharge) were consistent with findings documented in previous studies. ^1, 19, 31^ Furthermore, the observed variation in median Ct values across study periods indicates fluctuations in viral load and shedding intensity. The underlying factors contributing to these differences may include underlying study design differences, genotypes in circulation, changes in the laboratory protocols in use, among others. These observations warrant further investigation to better understand their implications for transmission dynamics. We also observed that samples with lower Ct values showed significantly higher sequencing success than those with higher Ct values. This has been reported previously ^48^ and is consistent with the inverse relationship between Ct value and viral load, ^49^ whereby lower Ct values reflect higher viral RNA concentrations, providing greater amounts of starting material for sequencing.

This study had limitations. First, hMPV genomic data was not generated for the period between 2018 and 2020 as the underlying studies did not collect samples during these years. This gap limited our capability to undertake a detailed longitudinal assessment of the temporal trends in genomic evolution and epidemiology of hMPV in the study population. Second, a high number of samples were excluded from the final analysis due to either non-amplification or suboptimal quality of recovered genome assemblies. This may have been caused by either viral RNA degradation due to long-term sample storage or our in-house sequencing protocol requires further optimization to sequence locally circulating hMPV strains, which may differ from the global genotypes used in the primer design.

In conclusion, we provide an update on the genomic epidemiology of hMPV in Kilifi, coastal Kenya (2016-2017 and 2021-2024), enriching the global hMPV genomic database and our understanding of locally circulating strains. Lineage replacement was observed over the years with the recent epidemic dominated by the A2c-wild-type sub-lineage and the documentation of a case of A2c-111-nt-dup strain in early 2021. Sustained genomic surveillance is needed to detect new variants in circulation and determine their significance to existing molecular diagnostics and infection clinical epidemiology.

## Data Availability

The genomes generated in this study are publicly available in GenBank under accession numbers PX279635 to PX279688. The code and associated resources are publicly accessible in the Havard Dataverse repository under the DOI: https://doi.org/10.7910/DVN/DV9DCM. ^50^ Data are available under the terms of the Creative Commons Attribution 4.0 International license.
