# Supplementary material for: Molecular Epidemiology of Human Metapneumovirus in Kilifi, Coastal Kenya, 2016-2017 and 2021-2024
Source: Wellcome Open Res. 2026 Jul 2;11:106. Originally published 2026 Feb 12. [Version 2] doi: 10.12688/wellcomeopenres.25865.2 (PMC13555068; doi:10.12688/wellcomeopenres.25865.2)
Supplement: Peer review history

# Reviewer report: Reviewer response for version 2

**DOI:** 10.21956/wellcomeopenres.29222.r159919

**Published:** 2026-07-20

**Authors:** Dr. Shrikant Verma

**Affiliations:** 1 Era University, Lucknow, Uttar Pradesh, India

1. The authors are encouraged to include detailed inclusion and exclusion criteria in the Materials and methods section. This information is essential for ensuring methodological clarity, minimizing selection bias of the manuscript.

 2. The Discussion section requires further strengthening. The authors are encouraged to compare their findings with previous studies, address potential reasons for similarities or discrepancies, discuss the study's strengths and limitations, and highlight the clinical and research implications of the results.

 3. In Discussion section author write “Genotype analysis using whole genome, G and F gene sequences, yielded consistent results across all the methods. This has been reported elsewhere”.  Reported elsewhere? The sentence requires revision for clarity and scientific precision.

 4. The statistical methods are adequately described; however, the analysis appears to rely primarily on univariate comparisons. The authors should consider including appropriate multivariable analyses, where applicable, to adjust for potential confounding factors and strengthen the validity of their conclusions.

Is the work clearly and accurately presented and does it cite the current literature?

Yes

If applicable, is the statistical analysis and its interpretation appropriate?

Partly

Are all the source data underlying the results available to ensure full reproducibility?

Yes

Is the study design appropriate and is the work technically sound?

Yes

Are the conclusions drawn adequately supported by the results?

Yes

Are sufficient details of methods and analysis provided to allow replication by others?

Yes

Reviewer Expertise:

Molecular Biology and Genetics

I confirm that I have read this submission and believe that I have an appropriate level of expertise to confirm that it is of an acceptable scientific standard, however I have significant reservations, as outlined above.

# Reviewer report: Reviewer response for version 1

**DOI:** 10.21956/wellcomeopenres.28469.r147690

**Published:** 2026-03-24

**Authors:** Carla della Ventura

**Affiliations:** 1 University of Milan, Milan, Milan, Italy

This study analyzed 52 near-complete genomes of human metapneumovirus (hMPV) from coastal Kenya, significantly expanding the limited genomic data available for the region and Africa overall. The findings provide an updated view of local molecular epidemiology and highlight the potential of these sequences to improve diagnostic and sequencing strategies.

 The results showed continuous co-circulation of hMPV lineages A and B, with shifts in dominant sub-lineages over time, including recent predominance of the A2c wild-type strain. Notably, a strain carrying a 111-nucleotide duplication in the G gene was identified for the first time in Kenya. Phylogenetic analysis suggested multiple viral introductions and connections with strains from other continents, consistent with global transmission driven by human mobility.

 The study also confirmed that genotyping based on whole genome or partial genes (G and F) yields consistent results, which is useful when full genome sequencing is not feasible. Epidemiologically, infections were most common in children under five years of age and associated with typical respiratory symptoms. Lower Ct values were linked to higher sequencing success, reflecting higher viral loads.

 However, the study was limited by missing data between 2018 and 2020 and the exclusion of many samples due to poor sequencing quality, possibly related to RNA degradation or suboptimal protocols. Overall, the findings emphasize the importance of sustained genomic surveillance to monitor emerging variants and their implications for diagnostics and disease dynamics.

Is the work clearly and accurately presented and does it cite the current literature?

Yes

If applicable, is the statistical analysis and its interpretation appropriate?

I cannot comment. A qualified statistician is required.

Are all the source data underlying the results available to ensure full reproducibility?

Yes

Is the study design appropriate and is the work technically sound?

Yes

Are the conclusions drawn adequately supported by the results?

Yes

Are sufficient details of methods and analysis provided to allow replication by others?

Yes

Reviewer Expertise:

My research focuses on viruses, with a particular emphasis on reconstructing their genomic evolutionary history. I use genomic and phylogenetic approaches to study how viruses evolve, spread, and diversify over time, with the aim of better understanding their epidemiology and emergence.”

I confirm that I have read this submission and believe that I have an appropriate level of expertise to confirm that it is of an acceptable scientific standard.

# Reviewer report: Reviewer response for version 1

**DOI:** 10.21956/wellcomeopenres.28469.r147695

**Published:** 2026-02-28

**Authors:** Stephanie Goya

**Affiliations:** 1 University of Washington Medical Center, Seattle, Washington, USA

The manuscript provides important genomic data on hMPV circulation in Coastal Kenya and contributes valuable sequences from a region that remains underrepresented in global datasets. The integration of outpatient surveillance and community-based sampling strengthens the epidemiological scope, and the temporal comparison across multiple years allows meaningful insights into lineage replacement dynamics. However, some methodological and reporting aspects require clarification to enhance transparency and reproducibility.

 In the section "RNA extraction, cDNA synthesis and multiplex PCR", it is not clearly stated whether cDNA synthesis was performed using random primers or gene-specific primers. As the priming strategy can influence genome coverage and amplification bias, particularly in tiled-amplicon approaches, clarification would be important.

 The manuscript states that global whole-genome sequences with complete metadata were downloaded from GISAID on January 30, 2025. Can the authors specify the exact database name within GISAID from which sequences were downloaded? In addition, the Discussion refers to potential bias related to sequence availability in GenBank; however, the authors do not indicate that GenBank was searched or included in the dataset. 

 In Table 2, the superscript “1 n(%)” is included in the footnote, apparently referring to the ResViRe column, but the table does not clearly indicate how this superscript applies. 

 In Table 2, because continuous variables were analyzed using the Kruskal–Wallis rank sum test, it would be more appropriate to report medians with interquartile ranges rather than medians with ranges, as IQR better reflects dispersion in non-normally distributed data. For variables with statistically significant Kruskal–Wallis results, post-hoc pairwise comparisons (e.g., Dunn's test with correction for multiple testing) would strengthen the interpretation by identifying which specific groups differ. For the age group comparisons analyzed using Pearson's Chi-square test, the authors should confirm that expected cell count assumptions were met, if not, a Fisher's exact test (or Monte Carlo simulation) would be more appropriate.

 Regarding Figure 3, in addition to presenting the absolute number of positive cases per month, it would be informative to include the monthly positivity rate using the total number of samples tested as the denominator. This would allow a clearer interpretation of temporal trends and better distinguish changes in viral circulation from fluctuations in sampling intensity.

 Finally, the interpretation of the 180-nt and 111-nt duplications in the G gene would benefit from engagement with recent literature (DOI: 10.1016/j.jcv.2025.105848, 10.64898/2026.01.18.26344359) suggesting that the 111-nt variant may not represent an independent duplication event but rather a derivative of the 180-nt duplication through a 69-nt deletion. The authors are encouraged to discuss how their phylogenetic and genomic findings align with or differ from these recent evolutionary hypotheses

Is the work clearly and accurately presented and does it cite the current literature?

Yes

If applicable, is the statistical analysis and its interpretation appropriate?

Partly

Are all the source data underlying the results available to ensure full reproducibility?

Yes

Is the study design appropriate and is the work technically sound?

Yes

Are the conclusions drawn adequately supported by the results?

Yes

Are sufficient details of methods and analysis provided to allow replication by others?

Partly

Reviewer Expertise:

Viral genomics, epidemiology, surveillance, evolution

I confirm that I have read this submission and believe that I have an appropriate level of expertise to confirm that it is of an acceptable scientific standard, however I have significant reservations, as outlined above.

## References

[1] : Phylodynamics of human metapneumovirus and evidence for a duplication-deletion model in G gene variant evolution. *Journal of Clinical Virology* .2025;180: 10.1016/j.jcv.2025.105848 10.1016/j.jcv.2025.105848 40769077

[2] : Genomic surveillance of human metapneumovirus in the United States, 2010 – 2025. *medRxiv* .2026; 10.64898/2026.01.18.26344359 10.64898/2026.01.18.26344359 PMC1360037042089368

# Response

**Published:** 2026-04-21

**Authors:** Dorcas Okanda

**Affiliations:** Epidemiology and Demography, Centre for Geographic Medicine Research Coast, Kilifi, Kilifi County, Kenya

**Question**: In the section "RNA extraction, cDNA synthesis and multiplex PCR", it is not clearly stated whether cDNA synthesis was performed using random primers or gene-specific primers. As the priming strategy can influence genome coverage and amplification bias, particularly in tiled-amplicon approaches, clarification would be important.

**Response**: cDNA synthesis was performed using the LunaScript RT Super Kit, which employs a random priming strategy. We have updated the manuscript to make this clear.

**Question**: The manuscript states that global whole-genome sequences with complete metadata were downloaded from GISAID on January 30, 2025. Can the authors specify the exact database name within GISAID from which sequences were downloaded? In addition, the Discussion refers to potential bias related to sequence availability in GenBank; however, the authors do not indicate that GenBank was searched or included in the dataset. 

**Response**: We apologise for the confusion caused. The database we searched and obtained the comparison global dataset is GenBank, accessed via the NCBI Virus portal. We have updated the Methods section to make this clear. No data from GISAID is included here.

**Question**: In Table 2, the superscript “1 n(%)” is included in the footnote, apparently referring to the ResViRe column, but the table does not clearly indicate how this superscript applies. 

**Response**: Thank you for this important clarification. We have revised the footnote of Table 2 to remove the ambiguous superscript reference and to clearly specify the data presentation format. The footnote now reads: “Median (IQR) reported for continuous variables; n (%) for categorical variables.”.  

**Question**: In Table 2, because continuous variables were analyzed using the Kruskal–Wallis rank sum test, it would be more appropriate to report medians with interquartile ranges rather than medians with ranges, as IQR better reflects dispersion in non-normally distributed data. For variables with statistically significant Kruskal–Wallis results, post-hoc pairwise comparisons (e.g., Dunn's test with correction for multiple testing) would strengthen the interpretation by identifying which specific groups differ. For the age group comparisons analyzed using Pearson's Chi-square test, the authors should confirm that expected cell count assumptions were met, if not, a Fisher's exact test (or Monte Carlo simulation) would be more appropriate.

**Response**: Thank you for these helpful suggestions. We have re-run the statistical analyses  suggestions and have updated Table 2  as follows: 1. **IQR reporting: **Continuous variables (Ct values and age) are now reported as median with interquartile range (Q1, Q3) instead of minimum–maximum values in Table 2, to better reflect data dispersion. 2. **Post-hoc analysis: **Following significant Kruskal–Wallis test results (p < 0.001 for both age and Ct values), we performed post-hoc pairwise comparisons using Dunn’s test with Bonferroni correction. These analyses show that the 2016–2017 period differs significantly from both later periods, while no significant differences were observed between 2021–2022 and 2023–2024. All results are now presented in **Table 3** and reflected in the Results section. 3. **Chi-square assumptions: **For age group comparisons, we confirmed that all expected cell counts met Chi-square assumptions (minimum expected count = 5.37). Therefore, Pearson’s Chi-square test was deemed appropriate, and the results (p < 0.001) are reported in the manuscript. We have updated Table 2 and added Table 3 to provide full transparency of the post-hoc comparisons with adjusted p-values.

**Question**: Regarding Figure 3, in addition to presenting the absolute number of positive cases per month, it would be informative to include the monthly positivity rate using the total number of samples tested as the denominator. This would allow a clearer interpretation of temporal trends and better distinguish changes in viral circulation from fluctuations in sampling intensity.

**Response**: Thank you for this suggestion. We have updated Figure 3 accordingly.

**Question**: Finally, the interpretation of the 180-nt and 111-nt duplications in the G gene would benefit from engagement with recent literature (DOI: 10.1016/j.jcv.2025.105848, 10.64898/2026.01.18.26344359) suggesting that the 111-nt variant may not represent an independent duplication event but rather a derivative of the 180-nt duplication through a 69-nt deletion. The authors are encouraged to discuss how their phylogenetic and genomic findings align with or differ from these recent evolutionary hypotheses

**Response:** We thank the reviewer for this comment and for highlighting this ongoing discussion. The hypothesis that 111-nt duplication is actually a derivative of the 180-nt duplication via a 69-nt deletion is thought provoking and adds valuable perspective to the interpretation of the emergence of these variants. Our study detected strains with only the 111-nt duplication. Due to a limited representative dataset (temporally and spatially) our analyses lacks the resolution to suggest the directionality of these events. We note that the 111-nt duplication was reported later in the literature than the 180-nt duplication, and the significant genomic surveillance gaps for hMPV globally. We have added a statement in the discussion highlighting this ongoing debate on the possibility of a deletion-derived origin of the 111-nt variant from 180-nt-duplication and the current limitations in conclusively resolving this relationship. We encourage generation of  broader datasets to better understand the evolutionary dynamics of these duplications and more generally hMPV evolutionary dynamics.
